# Effects of Water Deficit Irrigation on Phenolic Composition and Antioxidant Activity of Monastrell Grapes under Semiarid Conditions

**DOI:** 10.3390/antiox10081301

**Published:** 2021-08-18

**Authors:** Eva P. Pérez-Álvarez, Diego S. Intrigliolo, María Pilar Almajano, Pilar Rubio-Bretón, Teresa Garde-Cerdán

**Affiliations:** 1 Grupo VIENAP, Instituto de Ciencias de la Vid y del Vino (CSIC), Universidad de La Rioja, Gobierno de La Rioja, Ctra. de Burgos, Km. 6, 26007 Logroño, Spain; rubio_pilar@hotmail.com; 2 Centro de Edafología y Biología Aplicada del Segura (CEBAS), Campus Universitario de Espinardo, Ed. 25, 30100 Murcia, Spain; diego.intrigliolo@csic.es; 3 Centro de Investigación Sobre Desertificación (CSIC-UV-GV), Carretera CV-315, Km 10.7, 46113 Moncada, Spain; 4 Chemical Engineering Department, Universitat Politècnica de Catalunya, Av. Diagonal, 647, 08028 Barcelona, Spain; m.pilar.almajano@upc.edu

**Keywords:** phenolic compounds, antioxidant capacity, regulated deficit irrigation, ABTS, DPPH, ORAC, anthocyanins, flavonols, flavanols, non-flavonoids

## Abstract

The high phenolic compound content of grapes makes them an important source of natural antioxidants, among other beneficial health properties. Vineyard irrigation might affect berry composition and quality. Regulated deficit irrigation (RDI) is a widely used strategy to reduce the possible negative impact of irrigation on grapes, improving grape composition and resulting in water savings. Monastrell grapevines (*Vitis vinifera* L.) grown in eastern Spain were subjected to two water regime strategies: rainfed (non-irrigation) and RDI. The content of anthocyanins, flavonols, flavanols, hydroxybenzoic and hydroxycinnamic acids, and stilbenes was determined by HPLC and was related with total phenolic content and three antioxidant activity methods (ABTS, DPPH, and ORAC). The study aimed to evaluate and compare the phenolic composition and antioxidant potential of Monastrell grapes. The rainfed regime concentrated grapes in terms of phenolic compounds. Thus, total content of anthocyanins, flavonols, flavanols, hydroxybenzoic acids, and total phenols were higher in the rainfed grapes than in the RDI ones. Besides, the rainfed grapes doubled their antioxidant potential with respect to the RDI grapes with the ORAC method. Total phenolic content and antioxidant activity by ORAC assay positively correlated with most of the total phenolic compounds analyzed. This study demonstrates how field practices can modulate final grape composition in relation to their antioxidant activity.

## 1. Introduction

Phenolic compounds are among the most important and studied grape compounds, both for their contribution to the sensory properties of grapes and wines and for their antioxidant, antibacterial, anti-inflammatory, hypoglycemic, and hypolipidemic activities that have a great impact on human health [1,2].

Among other reactive species and as a consequence of the oxidative stress that reactive oxygen species (ROS) cause, they provoke biomolecular damage to cells and tissues of the living organisms [3]. The pathophysiology of many diseases, such as cancer, atherosclerosis, cardiovascular, metabolic and neurodegenerative diseases, and brain ageing is related to this oxidative damage [3,4]. It has been found that grape polyphenols reduce ROS levels, which decrease chronic inflammation and modulate inflammatory pathways, which has the potential to overcome chronic inflammation leading to the development of chronic diseases such as neurodegenerative diseases, Alzheimer’s disease, cancer, diabetes, cardiovascular disease, lung disease, arthritis, and autoimmune diseases [4]. Moreover, authors such as Sureda et al. [5] and García-Flores et al. [6] suggested that the effects of ROS during intense exercise observed in athletes of different disciplines can be reduced by the antioxidant potential beneficial of the fruit polyphenols.

Grapes are the fourth most produced fruit worldwide [2] and have a high content of polyphenols and an important nutritional value. In grapes, two main groups of phenolic compounds can be found: flavonoids (i.e., anthocyanins, flavanols, and flavanols) and non-flavonoids (i.e., hydroxycinnamic and hydroxybenzoic acids and stilbenes) [7]. Several factors, such as grape variety and maturity [8,9], genetic diversity [10], viticulture management [11], soil characteristics [12], environmental stress [13], grapevine health status [14], and winemaking conditions, modulate grapes’ phenolic profile and concentration. Besides, Pérez-Magariño and González-San José [15], reported that differences in viticultural management and enological techniques, harvest maturity, and grape variety modify the antioxidative properties, in quality and quantity, of phenolic compounds.

On the other hand, grapevines are often grown in semi-arid regions characterized by dry and warm summers [16]. In vitiviniculture, water management via irrigation has several implications on grape composition and, therefore, on wine quality [17]. Because water resources will be increasingly limited as a result of the climate change, to apply efficient deficit irrigation (DI) strategies will be more necessary [18]. Regulated deficit irrigation (RDI) is a promising, widely used technique that, with moderate annual volumes of water restricted below the full evapotranspiration (ETc) of a vineyard, allows to improve the concentration of grapes’ secondary metabolites, which confer important sensory attributes to grapes and wines [18]. However, the timing and the severity of the RDI strategy affects plant physiology, yield, and the metabolism of grapes in diverse ways [19]. Moreover, RDI effects are often cultivar-dependent [16].

Different authors have investigated the phenolic content and antioxidant capacity of grape by-products, especially grape juice, pomace, wine, and raisin [20,21,22,23,24], and more scarcely, fresh grape [25]. However, this is the first information about the relationship between the phenolic content and antioxidant capacity of Monastrell grapes under rainfed and RDI watering regimes. Therefore, the aim of the study was to evaluate and compare the polyphenol content and antioxidant activity (evaluating the radical scavenging according to three different methods: ABTS, DPPH, and ORAC) of the Monastrell grapes under rainfed and RDI watering regimes. Thus, the most important family groups of phenolic compounds on grape and grape extract were characterized in terms of antioxidant activity through chemical (ABTS, DPPH, and ORAC) determinations.

## 2. Materials and Methods

### 2.1. Vineyard Conditions and Grape Sampling

The study was carried out with cv. Monastrell (*Vitis vinifera* L.) grapes from a commercial vineyard located in the municipal area of Fuente-Álamo (Lat: 38°43′ N, Long: 1°28′ W, elevation 820 m a.s.l.), Albacete, in the southeast of Spain. Two watering treatments were applied: rainfed, where plants were not irrigated, and regulated deficit irrigation (RDI), where water application was carried out to optimize its use, taking into account the water needs of the plant. Thus, for the RDI system, the soil water balanced method proposed by FAO was used [26], and crop evapotranspiration (ETc) was estimated using the ETc = ETo × Kc formula, where ETo is the reference evapotranspiration calculated daily with the Penman–Monteith equation FAO56 [26] by a nearby meteorological station, and Kc is the crop coefficient. The water was applied through a drip irrigation system with one emitter of 3.8 L/h for each linear meter of pipe (two emitters per plant).

The experiment design consisted in a four replicate complete randomized design with four rows of grapevines for each replicate. Each row had twelve grapevines per treatment and surrounding perimeter grapevines were used as buffers. Then, grapes from 24 grapevines per each replicate of each treatment were used in the assay.

At harvest day, when grapes reached an average Brix between 24 and 24.5, a random set of 200 grapes per replicate was collected and frozen at −20 °C to analyze their phenolic composition.

### 2.2. Extraction of Grape Phenolic Compounds and Determination of Phenolic Composition by HPLC

Phenolic compounds were analyzed according to the method of Portu et al. [27]. A liquid chromatograph HPLC (Agilent Technologies, Palo Alto, CA, USA) equipped with a photodiode array detector (DAD) was used. Previously, grape phenolic compounds were extracted from 50 g of each frozen grape sample with 50 mL of a mixture of methanol/water/formic acid (50:48.5:1.5, *v/v/v*) according to the same author’s method. Briefly, the mixture was homogenized by Ultra-Turrax T-18 (IKA, Staufen, Germany) at 18,000 rpm for 1 min. The smooth pastes obtained were macerated for 10 min in an ultrasonic bath (JP Selecta, Barcelona, Spain) and centrifuged at 5000 rpm and 10 °C for 10 min. The supernatant was separated, and the resulting pellet was extracted up to three times using 50 mL of the solvent mixture each time. The supernatants were then combined, and the volume was annotated. Samples were transferred to vials and stored at −20 °C until beginning their determination.

Then, following Castillo-Muñoz et al. [28], the chromatographic conditions were: 10 µL of grape extract were injected into the chromatograph. In addition, isolation of non-anthocyanin compounds was performed based on Castillo-Muñoz et al. [29] method. Phenolic compounds were identified according to the retention times of pure compounds and the UV-Vis data obtained from authentic standards. For quantification, different wavelengths for each family of phenolic compounds (anthocyanins: 520 nm, flavonols: 360 nm, hydroxybenzoic and hydroxycinnamic acids and stilbenes: 320 nm, and flavanols: 280 nm) were used to extract the DAD chromatograms. Besides, the calibration graphs of the respective standards (*R*^2^ > 0.999) were done. When no standard was available, quantification was performed according to the calibration graph of the most similar compound [27,28]. Briefly, for anthocyanins, malvidin-3-*O*-glucoside was used; for flavonols, quercetin-3-*O*-glucoside was used; for free hydroxycinnamic acids and the corresponding tartaric esters, *trans*-caftaric acid was used; for procyanidins B1 and B2, catechin was used; for epigallocatechin, epicatechin was used; and for the *trans*- and *cis*-piceid and *trans*- and *cis*-resveratrol, their respective *trans* isomers were used.

The treatments were performed in quadruplicate, so the results for phenolic compounds in grapes correspond to the average of four analyses (*n* = 4) and concentrations in samples were expressed as milligrams per fresh weight of grape (mg/kg).

### 2.3. Determination of Total Phenolic Content (TPC)

Total Phenolic Content was determined by the Folin-Ciocalteu method described by Mosca [30]. The grape extract samples (explained in Section 2.2) and reagents were pipetted into each well in a 96-well microplate in this order: sample (adequately diluted), Folin-reagent (0.62 M), sodium carbonate solution 4% (*w/w*), and ultrapure water (1:4:4:4). After incubation in the dark for one hour at 25 °C, absorbance was measured at λ = 765 nm using a Fluostar Omega microplate reader (BMG-LabTech, Ortenberg, Germany). Gallic acid (GA) was used as standard (in the range 0.12 to 1.70 mM), and the results were expressed in mg GA Equivalents (mg GAE)/mL sample ± standard deviation. Each sample was analyzed in triplicate.

### 2.4. Determination of Free Radical Scavenging

The free radical scavenging activities of the grape extracts were determined using the 2,2-diphenyl-1-picryl-hydrazyl-hydrate (DPPH) radical test; 2,2′-Azobis(2-amidinopropane) dihydrochloride (AAPH) radical test (Oxygen Radical Absorbance Capacity (ORAC)); and 2,2′-Azino-bis(3-ethylbenzthiazoline-6-sulfonic acid) (ABTS) radical test (Trolox Equivalent Antioxidant Capacity, TEAC) according to the methods described below.

#### 2.4.1. DPPH Assay

The capacity of grape extract samples (explained in Section 2.2) to scavenge DPPH radicals was performed by the method of Villasante et al. [31]. The reagent, DPPH (0.1 mM in methanol), was added with a multi-channel-pipette to each well in a 96-well microplate. Initial absorbance was determined at λ = 517 nm at 37 °C using a Fluostar Omega microplate reader (BMG-LabTech, Ortenberg, Germany). Thereafter, an appropriate dilution of samples was added (proportion 0.1 sample: 1 reagent) and absorbance was measured 15, 30, 60, and 75 min later. Trolox^®^ standards were used and a calibration line of % inhibition against Trolox concentration (0.02–0.5 mM) was plotted. Results were expressed in mmol Trolox Equivalent (TE)/mL sample ± standard deviation. The measurements were done in triplicate for each sample.

The inhibition percentage was calculated using the following equation:

$$\%\mathrm{inhibition}\;\mathrm{of}\;\mathrm{sample}=\left(\frac{A_{0}-A_{s}}{A_{0}}\right)\times 100$$

where A_0_ = initial absorbance of DPPH solution; A_s_ = sample absorbance at the end of the reaction.

#### 2.4.2. ORAC Assay

The radical scavenging activity vs. AAPH was determined by the ORAC assay as described by Azman et al. [32]. Fluorescein in phosphate buffer solution (PBS) (10 mM, pH = 7.4) and grape extract samples (explained in Section 2.2.) adequately diluted (or standard or PBS) were pipetted into each well in a 96-well microplate. An initial fluorescence reading was recorded with λ_excitation_ = 485 nm and λ_emission_ = 520 nm at 37 °C using a Fluostar Omega microplate reader (BMG-LabTech, Ortenberg, Germany). Then, APPH (240 mM) was added, and fluorescence was recorded every 2 min for 2 h. The proportion of each compound was 6:1:1 (fluorescein:sample:AAPH). Trolox^®^ was used as standard (200 µM–12.5 mM) instead of sample and PBS blank was also determined. ORAC values were expressed as µmol TE/mL sample ± standard deviation. Each sample was measured in triplicate.

Area under the curve (AUC) and AUC_net_ were calculated using the following equations:

$$\mathrm{AUC}=\left(\frac{0.5+\sum_{i}^{n}f}{f_{o}}\right)\cdot 2;{\;\mathrm{AUC}}_{\mathrm{net}}={\mathrm{AUC}}_{s}-{\mathrm{AUC}}_{\mathrm{blank}}$$

where f = fluorescence at a given time; f_o_ = initial fluorescence; *n* = number of cycles; and 2 is the time (in min) of each cycle.

#### 2.4.3. TEAC Assay

The radical scavenging ability of grape extract samples (explained in Section 2.2) against ABTS radical cation was determined using a method based on that described by Segovia et al. [33]. The ABTS^•+^ was generated by mixing ABTS solution (7 mM, final concentration) with a potassium peroxodisulfate solution (2.45 mM, final concentration) and storing it in the dark overnight. The running ABTS solution was prepared by diluting it in PBS 1:100, reaching an absorbance value between 0.72 and 0.8 at λ = 734 nm. ABTS solution was pipetted into the well in a 96-well microplate and the initial value was read at 30 °C in a Fluostar Omega microplate reader (BMG-LabTech, Ortenberg, Germany). Then, the samples (1:10 proportion) were added, stirred, and incubated for 10 min and the final absorbance was measured. The calibration line was prepared with Trolox^®^ (0.021–0.55 mM) and the TEAC value is expressed as mmol Trolox Equivalent (TE)/mL ± standard deviation. Each sample was analyzed in triplicate.

### 2.5. Statistical Analysis

Significant differences for each parameter were assessed using a variance analysis (ANOVA) procedure. When the ANOVA test was significant, differences between means of the samples were compared using Duncan’s Multiple Range test (*p* < 0.05). A multiple factor analysis was also performed. SPSS Statistics Version 21.0 package for Windows (IBM, Chicago, IL, USA) was the software used for the statistical analysis.

## 3. Results and Discussion

### 3.1. Flavonoid Composition of Monastrell Grapes from Grapevines under Non-Irrigated (Rainfed) and Regulated Deficit Irrigation (RDI) Watering Regimes

#### 3.1.1. Anthocyanins

Table 1 shows the watering regime effect on the content of the five monomeric anthocyanin structures identified in the grape samples (3-*O*-glucosides (3-glc) of delphinidin, cyanidin, petunidin, peonidin and malvidin), their acetylated (3-acglc) and *trans*-*p*-coumaroylated (3-cmglc) derivates, and the *cis*- and *trans*-*p*-coumaroyl (cis-3-cmglc, trans-3-cmglc) and the caffeoyl (3-cfglc) derivates of malvidin. Most of these compounds showed a higher content in grapes from the rainfed treatment compared to those that were irrigated. As other authors have also found [12,34], malvidin-3-glc was the major anthocyanin compound in the grapes. Besides, the content of total non-acylated anthocyanins accounted for 83% of the total anthocyanins in grapes and total acylated anthocyanins accounted for about 16% (Table 1).

#### 3.1.2. Flavonols

The flavonol glycosides of the six flavonoid structures present in *Vitis vinifera* grapes are shown in Table 2. In this case, the watering regime only affected the content of myricetin-3-glcU, total myricetins, laricitrin-3-glc, ishorhamnetin-3-gal, and syringetin-3-glc, being always higher their content in rainfed grapes than in RDI. The quercetin flavonoid structure was the most abundant in Monastrell grapes, followed by the myricetins-type flavonols (Table 2). In Tempranillo grapes, Portu et al. [27] and Garde-Cerdán et al. [34] found these proportions changed, with flavonol-myricetin structures being in the majority, followed by those of the quercetin type. Zarrouk et al. [35] reported that the heat stress promotes the reduction in the UPD-glucose:flavonoid 3-*O*-glucosyltransferase activity, increasing the flavonol content. As the grapevines under the rainfed treatment had less vegetative growth (data not shown), the bunches were more exposed to the sun than in the RDI vines. Thus, the increase in anthocyanin and flavonol content observed in berries under the rainfed versus the RDI watering regime (since both family compounds share the same biosynthetic pathway) could be due to the effect of the higher temperature increase in the rainfed regime during grape ripening. In addition, yield was also lower in the rainfed vines (data not shown), facilitating the concentration of the analyzed compounds.

#### 3.1.3. Flavanols

Table 3 presents the concentrations of flavanols in Monastrell grapes under rainfed and RDI watering regimes. Only the content of epicatechin, the major flavanol in the grapes, and epicatechin-3-gallate increased with rainfed treatment with respect to RDI. When a water deficit strategy was used, Yu et al. [36] and Savoi et al. [37] also observed a limited or mild effect on flavanol accumulation in grapes.

### 3.2. Non-Flavonoid Composition of Monastrell Grapes from Grapevines under Non-Irrigated (Rainfed) and Regulated Deficit Irrigation (RDI) Watering Regimes

The concentration of non-flavonoid compounds determined in the Monastrell grapes from grapevines under non-irrigated and RDI water conditions are listed in Table 4. As occurs in other varieties, gallic acid was the majority non-flavonoid found in Monastrell samples. Only the content of total hydroxybenzoic acids and trans-fertaric acid was affected by the watering regime, increasing in grapes from rainfed grapevines with respect to those from the RDI strategy. There are few papers studying the effects of watering regimes on non-flavonoid compounds in grapes. Zarrouk et al. [35] reported an increase in sustained deficit irrigation (SDI) berries with respect to rainfed due to the attenuation of the negative effects of temperature on bunchers under the SDI regime.

### 3.3. Total Phenolic Compounds

**Figure 1:**
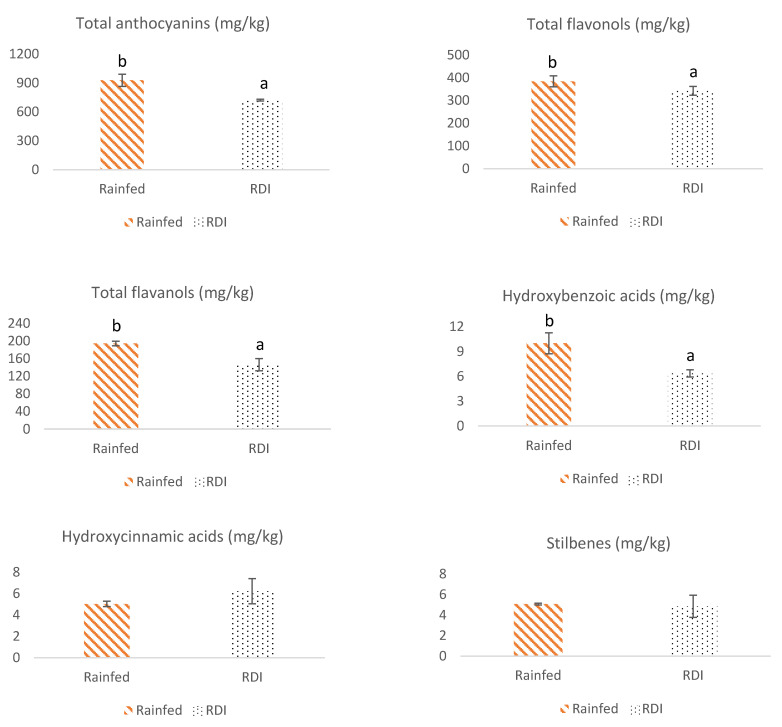

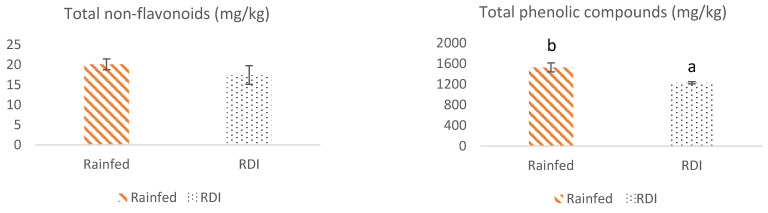
Mean values of total phenolic compounds and total content (mg/kg) of different groups of phenolic compounds in Monastrell grapes. Bars represent the standard deviations (*n* = 4). Different letters (a and b) indicate significant differences between grapes under non-irrigation (rainfed) and regulated deficit irrigation (RDI) regimes (*p* ≤ 0.05). When there is no difference between treatments (*p* > 0.05), no significance letters are shown.

The content of different groups of phenolic compounds in Monastrell grapes under non-irrigation (rainfed) and regulated deficit irrigation (RDI) watering regimes, as total anthocyanins, total flavonols, total flavanols, total hydroxybenzoic and hydroxycinnamic acids, total stilbenes, total non-flavonoids as well as total phenolic compounds, is shown in Figure 1. Thus, in general, grapes from the rainfed regime presented a higher content of total anthocyanins, total flavonols, total flavanols, total hydroxybenzoic acids and total phenolic compounds than the grapes from RDI. However, total hydroxycinnamic acids, total stilbenes, and total non-flavonoid content did not show significant differences between both irrigation regime grapes.

Anthocyanins are the most abundant phenolic compounds in Monastrell grapes, accounting for about 60% of the total of phenolic compounds. The anthocyanin degradation and/or the inhibition of its synthesis was reported when the clusters were exposed to high sunlight radiation [38] or/and high temperature [38,39]. Since both anthocyanin and flavonol compounds share the same biosynthetic enzyme, it can be suggested that the same factor that inhibited and/or degraded anthocyanins affects the flavonol accumulation. However, the impact of water availability on grape flavonols is controversial since there were studies such as those by Grimplet et al. [40] in which DI affected flavonols moderately, and another, i.e., Kennedy et al. [41], in which no effect of DI on these compounds was observed.

The increase of total phenolic compounds in rainfed grapes may be explained through the berry size, lower in rainfed (160.7 g/100 grapes) than in RDI (186.1 g/100 grapes). Thus, grape size indirectly affects the final concentration of phenolic compounds, and therefore, more diluted compounds could be found due to the rise of grape size and the skin to pulp ratio increase in the smaller grapes of grapevines subjected to water deficits [41,42,43]. Roby et al. [44] reported that grapes developed under water deficits had significantly more skin, the dominant tissue of flavonoid biosynthesis, than control grapes. Besides, authors such as Martínez-Lüscher et al. [45] and Luzio et al. [46] highlighted the importance of grape exposure to light, which genetically induced the biosynthesis of flavonoids. This means that the accumulation and composition of flavonoids in grapes are correlated by light quality and quantity exposure, which has a synergistic effect on the expression of genes in flavonoid biosynthesis pathway expression. The higher vigour observed in RDI grapevines (data not shown) could favor the higher presence of leaves with respect to the rainfed ones and consequently, due to the increase of the grapes’ shading, down-regulate flavonoid biosynthesis. As non-flavonoid compounds such as hydroxycinnamic acids and stilbenes are more abundant in grape pulp than flavonoid compounds, which are mainly found in the skin and seeds [47], it could be possible that in no-flavonoids content, the light exposure did not have such an effect, and no differences were observed between grapes from both watering regimes (Figure 1).

### 3.4. Antioxidant Capacity

The antioxidant potential of Monastrell grapes was assessed as ABTS, DPPH, and oxygen radical absorbance capacity (ORAC), three complementary antioxidant assays (Table 5). Total phenolic compounds (TPC) and the antioxidant tested by the ORAC method were higher in rainfed grapes than in RDI ones; meanwhile, grapes from the RDI regime had greater ABTS values than the rainfed ones. In the rainfed grapes, ORAC values almost doubled those of RDI grapes (Table 5).

The highest TPC in rainfed grapes can be explained by the smaller size in rainfed grapes compared to RDI. Thus, the phenolic compound concentration was higher in rainfed grapes. Grapes from rainfed treatment had the highest content of TPC and the strongest antioxidant capacity according to ORAC method. Thus, they may have greater health benefits than RDI grapes. In the ABTS method, RDI grapes are not significantly different from the rainfed ones (Table 5). In general, the scavenging ability of the different bioactive compounds varies widely by the mode of action of each radical [48], but it is more usual to find higher values of antioxidant capacity with ABTS than with the DPPH method [49], contrary to what was observed in our Monastrell grapes (Table 5). In Southern Portugal, Zarrouk et al. [50] observed higher total phenol content of the skin in RDI and non-irrigated Aragonez (syn. Tempranillo) vines than in conventional sustained DI ones during two consecutive years. However, although they reported an increasing trend of the ORAC activity in grape skin induced by RDI strategy, they suggested that other parameters other than phenolics (perhaps vitamins) are responsible for ORAC activity because, in their conditions, water deficits did not modify the bioactive quality of the grapes. Genebra et al. [51] suggested that ORAC activity increases until veraison and decreases thereafter. Thus, they supported the hypothesis of polyphenols oxidation during seed development reported by Kennedy et al. [52]. Besides, Genebra et al. [51] observed that the peak of ORAC activity is different depending on the grapevine water status, being maximal the ORAC activity at peak size stage in the seeds of non-irrigated grapes, while they were maximal at veraison for RDI and sustained DI ones. Thus, they corrobated previous results of Castellarin et al. [42,53] in which it was observed that water stress advanced the grapes’ ripening.

### 3.5. Relationship of Total Phenolic Content and Different Phenolic Groups According to Their Antioxidant Activity

#### 3.5.1. Total Polyphenol Content

**Figure 2:**
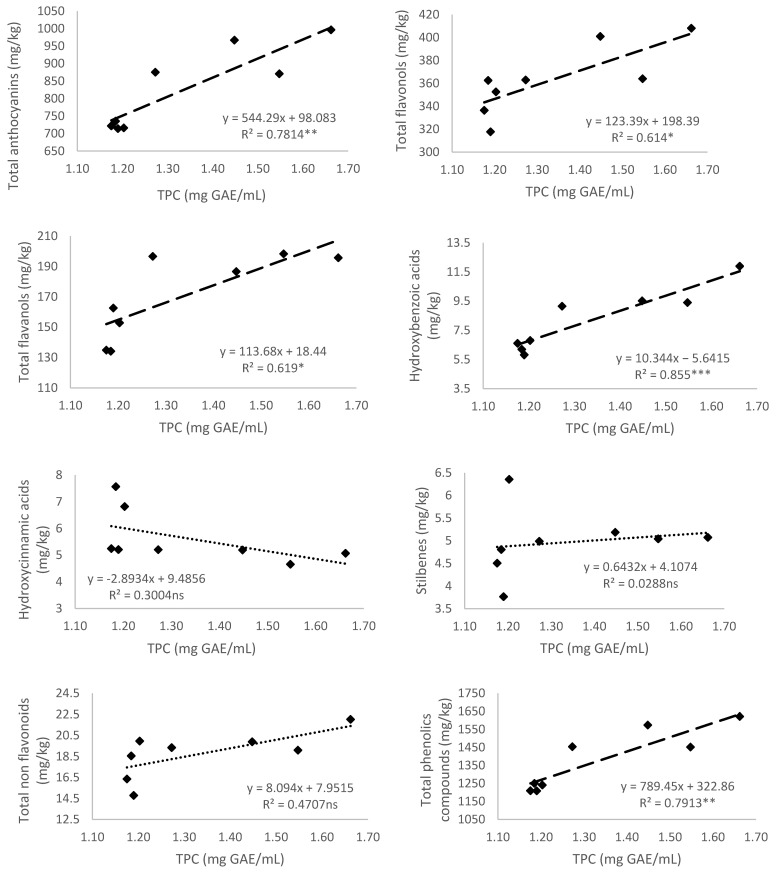
Relationship of the total phenolic content (TPC) (mg GAE/mL) and total anthocyanins, flavonols, flavanols, hydroxybenzoic and hydroxycinnamic acids, stilbenes, non-flavonoids, and content of total phenolic compounds (mg/kg) of Monastrell grapes. Values of the coefficient of determination (R^2^) are presented. Lines of linear regression are shown: (- - -) when relationships are significant (*p* < 0.05, *; *p* < 0.01, **; and *p* < 0.001, ***), or (⋯) when there are no significant differences (*p* > 0.05, ns).

The correlations between total polyphenol content (TPC) and the content of total phenolic groups were established in Figure 2. Total anthocyanins, total flavonols, total flavanols, total hydroxybenzoic acids, and total phenolic compounds had a high positive correlation with the values of TPC in Monastrell grapes under rainfed and RDI water status (Figure 2).

Li et al. [25] found a strong linear correlation between the total phenolic content and antioxidant activities in skins and pulps of eleven grape varieties, the Muscat Kyoto grapes being the richest in bioactive phenolic compounds. In their study, Tkacz et al. [54] found low correlations between antioxidant activity and each group of phenolic compounds of novel sea buckthorn-based smoothies. González-Tejedor et al. [55] reported opposite results, where the FRAP method best reflected the concentration of antioxidant compounds in a purple smoothie made from grape, cucumber, beet, and broccoli. Jara-Palacios et al. [56] observed that the antioxidant activity (measured by ORAC assay) in seeds, skins, stems, and pomace extracts of white Zamera cv. only agreed with total polyphenol content for skins and stems. However, for DPPH assay, the antioxidant activity was in agreement with the results of total polyphenol content for all the seeds, skins, stems, and pomace samples.

#### 3.5.2. Antioxidant Activity Measured by 2,2′-Azino-bis(3-ethylbenzthiazoline-6-sulfonic acid) (ABTS)

**Figure 3:**
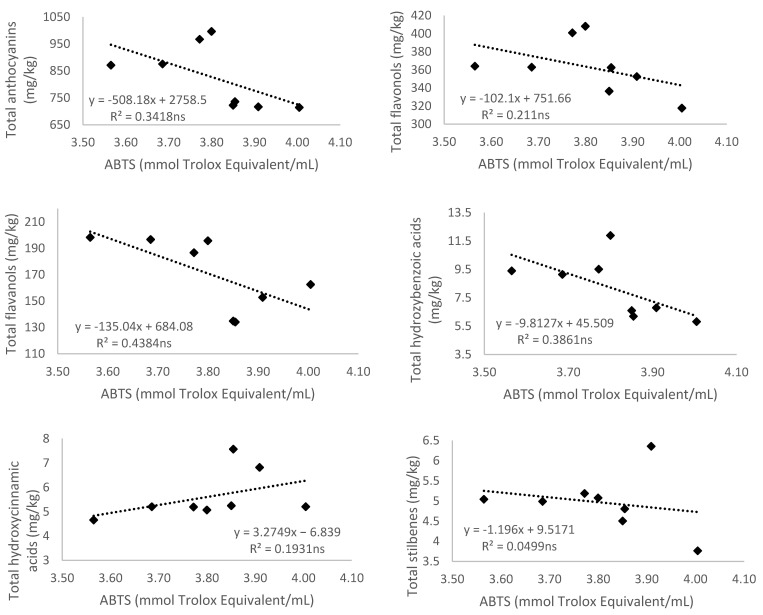

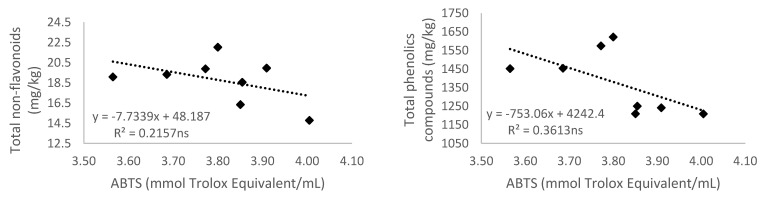
Relationship of the 2,2′-Azino-bis(3-ethylbenzthiazoline-6-sulfonic acid) (ABTS) (mmol Trolox/mL) and total anthocyanins, flavonols, flavanols, hydroxybenzoic and hydroxycinnamic acids, stilbenes, non-flavonoids, and content of total phenolic compounds (mg/kg) of Monastrell grapes. Values of the coefficient of determination (R^2^) are presented. Lines of linear regression are shown: there are no significant differences (*p* > 0.05, ns).

Correlations between ABTS values and the total of different groups of phenolic compounds were established (Figure 3). However, no significant relationships were found between the antioxidant activity measured with the ABTS method and the total of the different groups of phenolic compounds determined, nor with the total of all of them.

To the best of our knowledge, this is the first study relating the antioxidant activity measured as ABTS of Monastrell grapes under two different watering regimes and the different groups of phenolic compounds. In smoothies based on pome and berry fruits, Teleszko and Wojdyło [57] did not find correlations for ABTS with phenolic acids and flavonols. In grape wines, Wojdylo et al. [58] found that the reduction in the ABTS antioxidant capacity measured after fermentation and after maturation was related to a decrease in polyphenol content. They observed a positive correlation (*r* = 0.69) between the total phenolic compounds and ABTS antioxidant capacity. In their case, they found a positive correlation between anthocyanins, flavonols, flavan-3-ols content (*r* = 0.60, 0.64, and 0.66, respectively), and antioxidant capacity in Dornfelder cv. samples when ABTS assay was used.

#### 3.5.3. Antioxidant Activity Measured by 2,2-diphenyl-1-picryl-hydrazyl-hydrate (DPPH)

**Figure 4:**
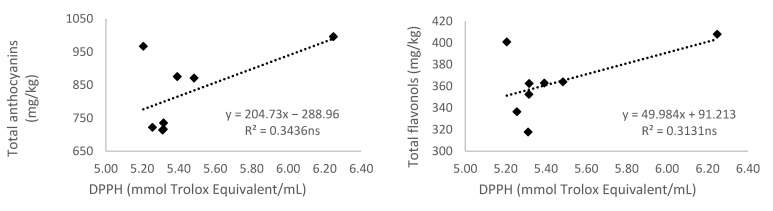

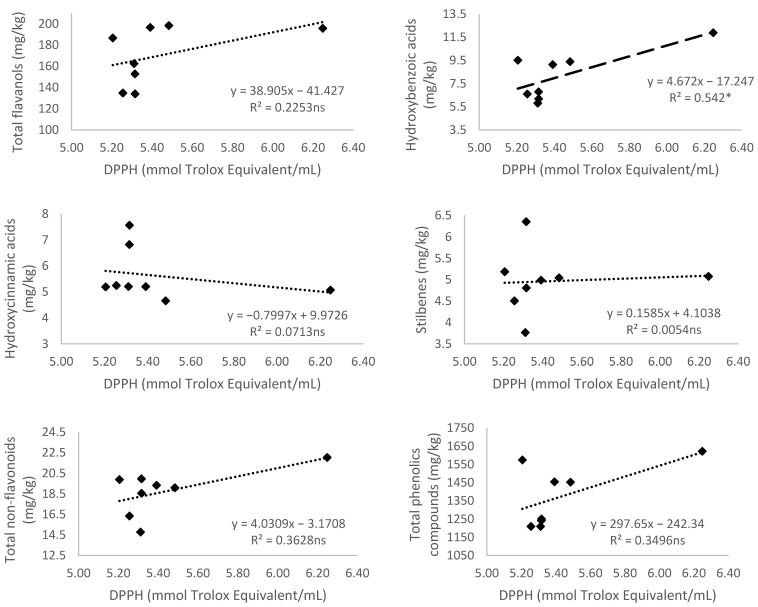
Relationship of the 2,2-diphenyl-1-picryl-hydrazyl-hydrate (DPPH) (mmol Trolox/mL) and total anthocyanins, flavonols, flavanols, hydroxybenzoic and hydroxycinnamic acids, stilbenes, non-flavonoids, and content of total phenolic compounds (mg/kg) of Monastrell grapes. Values of the coefficient of determination (R^2^) are presented. Lines of linear regression are shown: (- - -) when relationships are significant (*p* < 0.05, *), or (⋯) when there are no significant differences (*p* > 0.05, ns).

In the case of the relations between DPPH values and the total of different groups of phenolic compounds (Figure 4), only the total of hydroxybenzoic acids content showed a significant correlation (*r*^2^ = 0.542; *p* < 0.05). However, Karaman et al. [59] observed correlations among ABTS and DPPH antioxidant assays and total phenolic compounds and catechin content (in seeds, skins, and stems), total anthocyanin (in skins), *trans*-resveratrol (in seeds), and rutin (in stems) of six grapevine varieties grown in Turkey. They, as well as Xu et al. [60], suggested that both the ABTS and DPPH antioxidant methods are almost comparable and interchangeable in order to characterize the grape antioxidant capacities. Karaman et al. [59] also indicated that as the correlation between phenolic compounds and antioxidant capacity can be positive or negative, it is questionable, so further studies are needed. Tkacz et al. [54] found a low correlation between DPPH activity and bioactive compounds of novel sea buckthorn-based smoothies.

#### 3.5.4. Antioxidant Activity Measured by 2,2′-Azobis(2-amidinopropane) dihydrochloride (AAPH)—Oxygen Radical Absorbance Capacity (ORAC)

**Figure 5:**
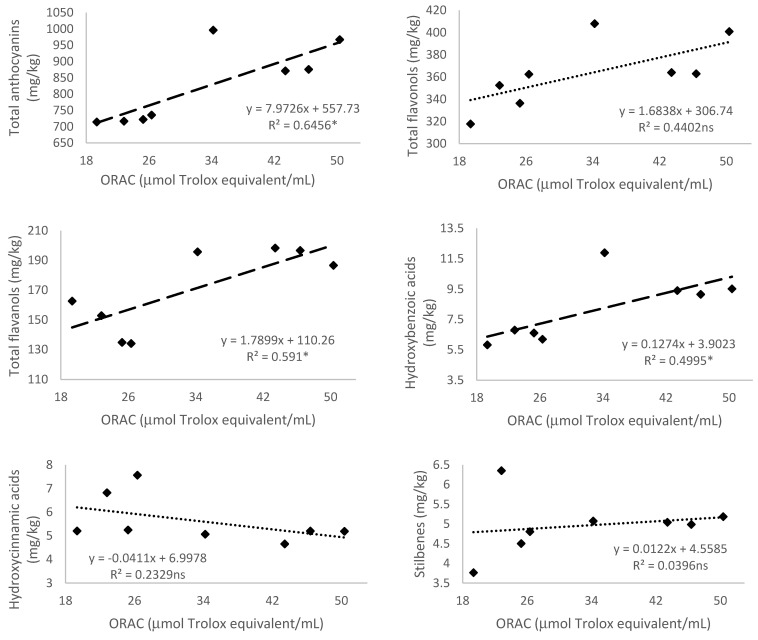

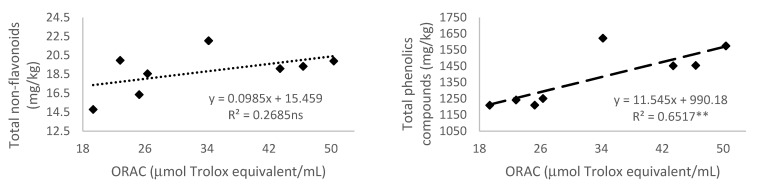
Relationship of the 2,2′-Azobis(2-amidinopropane) dihydrochloride (AAPH)—Oxygen radical absorbance capacity (ORAC) (μmol Trolox/mL) and total anthocyanins, flavonols, flavanols, hydroxybenzoic and hydroxycinnamic acids, stilbenes, non-flavonoids, and content of total phenolic compounds (mg/kg) of Monastrell grapes. Values of the coefficient of determination (R^2^) are presented. Lines of linear regression are shown: (- - -) when relationships are significant (*p* < 0.05, *; *p* < 0.01, ** or (⋯) when there are no significant differences (*p* > 0.05, ns).

The ORAC antioxidant activity method resulted in a strong correlation with the content of total anthocyanins, total flavanols, total hydroxybenzoic acids, and total phenolic compounds, achieving *r*^2^ = 0.646, 0.591, 0.499, and 0.652, respectively (*p* < 0.05) (Figure 5). Thus, this method for determining antioxidant activity had a higher correlation with the phenolic compounds measured in Monastrell grapes than ABTS and DDPH methods. Moreover, Nowicka et al. [61] in their studies with *Prunus*-fruit smoothies reported that the correlation between ORAC and phenolic compounds, including their groups, was higher than for ABTS. The ORAC value increased for sea buckthorn-based with apple, carrot, and parsley in a study by Tkacz et al. [54]. Jara-Palacios et al. [56] found the lowest ORAC values with ethanol extracts in seeds, skins, stems, and pomace of Zalema (*Vitis vinifera* L.) winemaking byproducts. However, they reported a higher scavenging capacity of peroxyl radicals (measured by ORAC assays) from the ethanol, ethanol/water, and water extracts from skins, than those from seeds. Matos et al. [62] reported a correlation (with R^2^ > 0.95) between the ORAC values and the relative amounts of flavonols for red wine lees, grape marc of Tempranillo, and Port wine lees. They observed that both TPC and the antioxidant activity values are highly variable and depend on different factors such as the grape variety, maturation stage, environmental conditions during grape growth, and vinifications, among others. Faria et al. [63] and Genebra et al. [51] reported that the ORAC correlated with all flavanol compounds, procyanidin dimer being the most antioxidative compound in different phenolics. Genebra et al. [51] observed that in sustained DI seeds at full maturation, the ORAC activity was significantly greater than in RDI and non-irrigation seeds. This suggested that the presence of peroxyl radicals in seeds from both DI treatments was affected by water availability which caused it to be enhanced, and this may have implications in winemaking, especially related to the bitterness and astringency of red wines.

## 4. Conclusions

In this assay, we found that the irrigation regime influences in a different way the content of phenolic compounds. The non-enzymatic antioxidant capacities were modulated by water deficit and correlated with phenolic compounds, which may be used for promoting health benefits. Thus, under semiarid climatic conditions, comparing grapes from Monastrell plants under a rainfed watering regime with others under an RDI strategy, higher contents of anthocyanins, flavonols, flavanols, hydroxybenzoic acids, and total phenolic compounds were obtained in rainfed grapes. The smaller size obtained in the rainfed grapes favored the concentration of these phenolic compounds; however, the total content of hydroxycinnamic acids, stilbenes, and total non-flavonoids were not affected. Besides, watering regime affected the total phenolic content and modulated the non-enzymatic antioxidant capacities (ABTS, DPPH, and ORAC). Antioxidant activities, especially ORAC assay, correlated positively with most groups of phenolic compounds found in grapes, except for the non-flavonoid content. The synergistic and antagonistic interactions that occur between phenolic compounds could explain the results of antioxidant activity obtained [64].

## Figures and Tables

**Table 1:** Individual anthocyanin content (mg/kg) in Monastrell grapes from grapevines under non-irrigated (rainfed) and regulated deficit irrigation (RDI) conditions.

	*Treatments* (*T*)		
*Anthocyanins*	Rainfed	RDI	*p*-Value ^1^
Delphinidin-3-glc	101.5 ± 18.5	86.1 ± 5.8	0.224
Cyanidin-3-glc	78.0 ± 5.7	72.3 ± 7.4	0.350
Petunidin-3-glc	108.9 ± 17.0	87.7 ± 4.0	0.088
Peonidin-3-glc	106.7 ± 5.6 b	93.4 ± 2.9 a	*
Malvidin-3-glc	370.1 ± 27.3 b	273.2 ± 2.7 a	**
***Total non-acylated***	**765.2 ± 60.4 b**	**612.6 ± 12.8 a**	***
Delphinidin-3-acglc	5.3 ± 0.4 b	4.1 ± 0.4 a	*
Cyanidin-3-acglc	3.3 ± 0.2	2.8 ± 0.3	0.437
Petunidin-3-acglc	5.0 ± 0.4 b	4.1 ± 0.2 a	**
Peonidin-3-acglc	2.4 ± 0.3	2.3 ± 0.1	0.203
**Malvidin-3-acglc**	**14.0 ± 0.8 b**	**10.4 ± 0.9 a**	**
Delphinidin-3-cmglc	10.9 ± 1.5 b	8.5 ± 1.1 a	*
Petunidin-3-cmglc	16.3 ± 2.3 b	10.9 ± 0.2 a	**
Cyanidin-3-cmglc	11.0 ± 0.1 b	10.4 ± 0.1 a	***
Peonidin-3-cmglc	19.0 ± 1.1 b	11.4 ± 0.5 a	***
Malvidin-3-cis-cmglc	3.6 ± 0.7	2.6 ± 0.1	0.203
Malvidin-3-trans-cmglc	68.9 ± 9.7 b	40.1 ± 1.7 a	**
Malvidin-3-cfglc	2.5 ± 0.3	2.0 ± 0.1	0.286
***Total acylated***	**162.3 ± 14.3 b**	**109.6 ± 3.2 a**	**

Abbreviations: glc: glucoside, acglc: acetylglucoside, cmglc: *trans*-*p*-coumaroylglucoside, cfglc: caffeoylglucoside. For each anthocyanin, different lowercase letters indicate significant differences between treatments (*p* ≤ 0.05). ^1^ Statistical significance: * *p* ≤ 0.05, ** *p* ≤ 0.01, and *** *p* ≤ 0.001. When there is no difference between treatments (*p* > 0.05), no significance letters are shown. Experiments were performed in quadruplicate, and results for each parameter presented are means ± standard deviation (*n* = 4).

**Table 2:** Flavonol content (mg/kg) in Monastrell grapes from grapevines under non-irrigated (rainfed) and regulated deficit irrigation (RDI) conditions.

	*Treatments* (*T*)		
*Flavonols*	Rainfed	RDI	*p*-Value ^1^
Myricetin-3-glcU	12.0 ± 1.8 b	6.5 ± 0.5 a	**
Myricetin-3-gal	8.8 ± 1.6	6.7 ± 1.0	0.066
Myricetin-3-glc	77.3 ± 8.2	68.4 ± 2.4	0.139
**Total myricetins**	**98.1 ± 7.9 b**	**81.5 ± 2.6 a**	**
Quercetin-3-glcU	101.9 ± 17.7	77.5 ± 4.5	0.080
Quercetin-3-glc	129.4 ± 14.4	140.4 ± 15.3	0.425
**Total quercetins**	**231.4 ± 16.7**	**218.0 ± 14.6**	**0.279**
**Laricitrin-3-glc**	**13.8 ± 0.8 b**	**9.0 ± 0.5 a**	***
Kaempferol-3-gal	3.1 ± 0.6	1.5 ± 0.3	0.067
Kaempferol-3-glc	25.0 ± 0.9	21.8 ± 4.8	0.297
**Total kaempferols**	**28.1 ± 0.6**	**23.3 ± 4.9**	**0.120**
Isorhamnetin-3-gal	2.2 ± 0.2 b	1.7 ± 0.1 a	*
Isorhamnetin-3-glc	4.2 ± 0.4	4.8 ± 0.6	0.519
**Total isorhamnetins**	**6.5 ± 0.4**	**6.2 ± 0.4**	**0.537**
**Syringetin-3-glc**	**6.0 ± 0.1 b**	**4.3 ± 0.2 a**	**

Abbreviations: glcU: glucuronide, gal: galactoside, glc: glucoside. For each flavonol, different lowercase letters indicate significant differences between treatments (*p* ≤ 0.05). ^1^ Statistical significance: * *p* ≤ 0.05, ** *p* ≤ 0.01, and *** *p* ≤ 0.001. When there is no difference between treatments (*p* > 0.05), no significance letters are shown. Experiments were performed in quadruplicate, and results for each parameter are presented as means ± standard deviation (*n* = 4).

**Table 3:** Flavanol content (mg/kg) in Monastrell grapes from grapevines under non-irrigated (rainfed) and regulated deficit irrigation (RDI) conditions.

	*Treatments* (*T*)		
Flavanols	Rainfed	RDI	*p*-Value ^1^
Catechin	48.1 ± 3.3	38.6 ± 8.1	0.131
Epicatechin	58.1 ± 1.1 b	37.6 ± 2.7 a	***
Epicatechin-3-gallate	68.8 ± 4.3 b	50.4 ± 5.8 a	**
Procyanidin B1	10.0 ± 1.4	8.8 ± 1.5	0.242
Procyanidin B2	9.5 ± 1.3	10.8 ± 1.2	0.322

For each flavanol, different letters indicate significant differences between treatments (*p* ≤ 0.05). ^1^ Statistical significance: ** *p* ≤ 0.01, and *** *p* ≤ 0.001. When there is no difference between treatments (*p* > 0.05), no significance letters are shown. Experiments were performed in quadruplicate, and results for each parameter are presented as means ± standard deviation (*n* = 4).

**Table 4:** Non-flavonoid content (mg/kg) in Monastrell grapes from grapevines under non-irrigated (rainfed) and regulated deficit irrigation (RDI) conditions.

	*Treatments* (*T*)		
*Non-Flavonoids*	Rainfed	RDI	*p*-Value ^1^
***Hydroxybenzoic acids***			
Syringic acid	3.9 ± 0.6	1.5 ± 0.2	0.058
Gallic acid	6.1 ± 1.4	4.9 ± 0.5	0.147
***Hydroxycinnamic acids***			
trans-Caftaric acid	1.5 ± 0.3	2.5 ± 0.8	0.132
trans + cis-Coutaric acids	1.0 ± 0.1	1.6 ± 0.4	0.116
Caffeic acid	1.0 ± 0.1	1.0 ± 0.1	0.489
trans-Fertaric acid	1.0 ± 0.1 b	0.9 ± 0.1 a	**
p-Coumaric acid	0.4 ± 0.1	0.4 ± 0.1	0.718
***Stilbenes***			
trans-Piceid	4.1 ± 0.2	3.9 ± 1.4	0.822
cis-Piceid	0.4 ± 0.1	0.4 ± 0.1	0.866
trans-Resveratrol	0.3 ± 0.1	0.3 ± 0.1	0.558
cis-Resveratrol	0.3 ± 0.1	0.2 ± 0.1	0.064

For each non-flavonoid, different letters indicate significant differences between treatments (*p* ≤ 0.05). ^1^ Statistical significance: ** *p* ≤ 0.01. When there is no difference between treatments (*p* > 0.05), no significance letters are shown. Experiments were performed in quadruplicate, and results for each parameter presented as means ± standard deviation (*n* = 4).

**Table 5:** Total phenolic content (TPC) and free radical scavenging activities (ABTS, DPPH, and ORAC) of Monastrell grapes under non-irrigation (rainfed) and regulated deficit irrigation (RDI) regime.

	TPC (mg GAE/mL)	ABTS ^1^ (mmol TE/mL)	DPPH (mmol TE/mL)	ORAC (μmol TE/mL)
Rainfed	1.5 ± 0.2 ^b^	3.7 ± 0.1 ^a^	5.6 ± 0.5	43.6 ± 6.9 ^b^
RDI	1.2 ± 0.1 ^a^	3.9 ± 0.1 ^a^	5.3 ± 0.1	23.4 ± 3.1 ^a^
*p*-value ^2^	**	*	0.265	**

^1^ ABTS: 2,2′-Azino-bis(3-ethylbenzthiazoline-6-sulfonic acid), DPPH: 2,2-diphenyl-1-picryl-hydrazyl-hydrate, ORAC: 2,2′-Azobis(2-amidinopropane) dihydrochloride (AAPH) radical test-Oxygen radical absorbance capacity (ORAC), GAE: galic acid equivalent, TE: Trolox equivalent. All parameters are given as average values ± the standard deviations (*n* = 4). Different letters (^a^ and ^b^) indicate significant differences between grapes under non-irrigation (rainfed) and regulated deficit irrigation (RDI) regime (*p* < 0.05). ^2^ Statistical significance: * *p* ≤ 0.05, ** *p* ≤ 0.01. When there is no difference between treatments (*p* > 0.05), no significance letters are shown.

## Data Availability

All data generated or analyzed during this study are included in this published paper.

## References

[1] Li L., Sun B. (2017). Grape and wine polymeric polyphenols: Their importance in enology. Crit. Rev. Food Sci. Nutr..

[2] Elejalde E., Villarán M.C., Alonso R.M. (2021). Grape polyphenols supplementation for exercise-induced oxidative stress. J. Int. Soc. Sports Nutr..

[3] Luo J., Mills K., Le Cessie S., Noordam R., Van Heemst D. (2019). Ageing, age-related diseases and oxidative stress: What to do next?. Ageing Res. Rev..

[4] Poprac P., Jomova K., Simunkova M., Kollar V., Rhodes C.J., Valko M. (2017). Targeting Free Radicals in Oxidative Stress-Related Human Diseases. Trends Pharmacol. Sci..

[5] Sureda A., Tejada S., Bibiloni M.D.M., Tur J.A., Pons A. (2014). Polyphenols: Well beyond the antioxidant capacity: Polyphenol supplementation and exercise-induced oxidative stress and inflammation. Curr. Pharm. Biotechnol..

[6] García-Flores L.A., Medina S., Gómez C., Wheelock C.E., Cejuela R., Martínez-Sanz J.M., Oger C., Galano J.-M., Durand T., Hernández-Sáez A. (2017). Aronia—Citrusjuice (polyphenol-rich juice) intake and elite triathlon training: A lipidomic approach using representative oxylipins in urine. Food Funct..

[7] Portu J., Santamaría P., Alfaro I.L., López R., Garde-Cerdán T. (2015). Methyl Jasmonate Foliar Application to Tempranillo Vineyard Improved Grape and Wine Phenolic Content. J. Agric. Food Chem..

[8] Fanzone M., Zamora F., Jofré V., Assof M., Neira P. (2011). Phenolic composition of Malbec grape skins and seeds from Valle de Uco (Mendoza, Argentina) during ripening. Effect of cluster thinning. J. Agric. Food Chem..

[9] Aubert C., Chalot G. (2018). Chemical composition, bioactive compounds, and volatiles of six table grape varieties (*Vitis vinifera* L.). Food Chem..

[10] Bustamante L., Sáez V., Hinrichsen P., Castro M.H., Vergara C., Von Baer D., Mardones C. (2017). Differences in Vvufgt and VvmybA1 Gene Expression Levels and Phenolic Composition in Table Grape (*Vitis vinifera* L.) ‘Red Globe’ and Its Somaclonal Variant ‘Pink Globe’. J. Agric. Food Chem..

[11] Garde-Cerdán T., Portu J., López R., Santamaría P. (2015). Effect of Foliar Applications of Proline, Phenylalanine, Urea, and Commercial Nitrogen Fertilizers on Stilbene Concentrations in Tempranillo Musts and Wines. Am. J. Enol. Vitic..

[12] Pérez-Álvarez E.P., Martínez-Vidaurre J.M., Garde-Cerdán T. (2019). Anthocyanin composition of grapes from three different soil types in cv. Tempranillo, A.O.C. Rioja vineyards. J. Sci. Food Agric..

[13] Ojeda H., Andary C., Kraeva E., Carbonneau A., Deloire A. (2002). Influence of pre- and postveraison water deficit on syn-thesis and concentration of skin phenolic compounds during berry growth of *Vitis vinifera* cv. Shiraz. Am. J. Enol. Vitic..

[14] Rusjan D., Veberic R., Mikulič-Petkovšek M. (2012). The response of phenolic compounds in grapes of the variety ‘Chardonnay’ (*Vitis vinifera* L.) to the infection by phytoplasma *Bois noir*. Eur. J. Plant Pathol..

[15] Perez-Magariño S., José M.L.G.-S. (2004). Evolution of Flavanols, Anthocyanins, and Their Derivatives during the Aging of Red Wines Elaborated from Grapes Harvested at Different Stages of Ripening. J. Agric. Food Chem..

[16] Kovalenko Y., Tindjau R., Madilao L.L., Castellarin S.D. (2020). Regulated deficit irrigation strategies affect the terpene accumulation in Gewürztraminer (*Vitis vinifera* L.) grapes grown in the Okanagan Valley. Food Chem..

[17] Gambetta G.A., Herrera J.C., Dayer S., Feng Q., Hochberg U., Castellarin S.D. (2020). The physiology of drought stress in grapevine: Towards an integrative definition of drought tolerance. J. Exp. Bot..

[18] Romero P., Botía P., del Amor F.M., Gil-Muñoz R., Flores P., Navarro J.M. (2019). Interactive effects of the rootstock and the deficit irrigation technique on wine composition, nutraceutical potential, aromatic profile, and sensory attributes under semiarid and water limiting conditions. Agric. Water Manag..

[19] Mirás-Avalos J.M., Buesa I., Llacer E., Bello M.A.J., Risco D., Castel J.R., Intrigliolo D.S. (2016). Water Versus Source–Sink Relationships in a Semiarid Tempranillo Vineyard: Vine Performance and Fruit Composition. Am. J. Enol. Vitic..

[20] Jacob J.K., Hakimuddin F., Paliyath G., Fisher H. (2008). Antioxidant and antiproliferative activity of polyphenols in novel high-polyphenol grape lines. Food Res. Int..

[21] Meng J., Fang Y., Zhang A., Chen S., Xu T., Ren Z., Han G., Liu J., Li H., Zhang Z. (2011). Phenolic content and antioxidant capacity of Chinese raisins produced in Xinjiang Province. Food Res. Int..

[22] De Castilhos M.B.M., Gómez-Alonso S., García-Romero E., del Bianchi V.L., Hermosín-Gutiérrez I. (2017). Isabel red wines produced from grape pre-drying and submerged cap winemaking: A phenolic and sensory approach. LWT Food Sci. Technol..

[23] Toscano L.T., Silva A.S., Toscano L.T., Tavares R.L., Biasoto A., de Camargo A.C., da Silva C.S.O., Gonçalves M.D.C.R., Shahidi F. (2017). Phenolics from purple grape juice increase serum antioxidant status and improve lipid profile and blood pressure in healthy adults under intense physical training. J. Funct. Foods.

[24] Fernández-Fernández A.M., De Hond A.I., Dellacassa E., Medrano A., Del Castillo M.D. (2019). Assessment of antioxidant, antidiabetic, antiobesity, and anti-inflammatory properties of a Tannat winemaking by-product. Eur. Food Res. Technol..

[25] Li F.-X., Yang Y.-X., Yin R., Ming J. (2019). Comparison of phenolic profiles and antioxidant activities in skins and pulps of eleven grape cultivars (*Vitis vinifera* L.). J. Integr. Agric..

[26] Allen R.G., Pereira L.S., Raes D., Smith M. (1998). Crop evapotranspiration—Guidelines for computing crop water requirements—FAO irrigation and drainage paper 56. Rome.

[27] Portu J., López R., Baroja E., Santamaría P., Garde-Cerdán T. (2016). Improvement of grape and wine phenolic content by foliar application to grapevine of three different elicitors: Methyl jasmonate, chitosan, and yeast extract. Food Chem..

[28] Castillo-Muñoz N., Fernández-González M., Gómez-Alonso S., García-Romero E., Hermosín-Gutiérrez I., García-Romero E. (2009). Red-Color Related Phenolic Composition of Garnacha Tintorera (*Vitis vinifera* L.) Grapes and Red Wines. J. Agric. Food Chem..

[29] Castillo-Muñoz N., Gómez-Alonso S., García-Romero E., Hermosín-Gutiérrez I. (2007). Flavonol Profiles of *Vitis vinifera* Red Grapes and Their Single-Cultivar Wines. J. Agric. Food Chem..

[30] Mosca F., Hidalgo G.I., Villasante J., Almajano M.P. (2018). Continuous or Batch Solid-Liquid Extraction of Antioxidant Compounds from Seeds of Sterculia apetala Plant and Kinetic Release Study. Molecules.

[31] Villasante J., Pérez-Carrillo E., Heredia-Olea E., Metón I., Almajano M.P. (2019). In Vitro Antioxidant Activity Optimization of Nut Shell (Carya illinoinensis) by Extrusion Using Response Surface Methods. Biomolecules.

[32] Azman N.A.M., Gallego M.G., Segovia F., Abdullah S., Shaarani S.M., Pablos M.P.A. (2016). Study of the Properties of Bearberry Leaf Extract as a Natural Antioxidant in Model Foods. Antioxidants.

[33] Segovia F.J., Corral-Pérez J.J., Almajano M. (2016). Avocado seed: Modeling extraction of bioactive compounds. Ind. Crop. Prod..

[34] Garde-Cerdán T., Gutiérrez-Gamboa G., Ayestarán B., González-Lázaro M., Rubio-Bretón P., Pérez-Álvarez E. (2020). Influence of seaweed foliar application to Tempranillo grapevines on grape and wine phenolic compounds over two vintages. Food Chem..

[35] Zarrouk O., Brunetti C., Egipto R., Pinheiro C., Genebra T., Gori A., Lopes C., Tattini M., Chaves M.M. (2016). Grape Ripening Is Regulated by Deficit Irrigation/Elevated Temperatures According to Cluster Position in the Canopy. Front. Plant Sci..

[36] Yu R., Cook M.G., Yacco R.S., Watrelot A., Gambetta G., Kennedy J.A., Kurtural S.K. (2016). Effects of Leaf Removal and Applied Water on Flavonoid Accumulation in Grapevine (Vitis viniferaL. cv. Merlot) Berry in a Hot Climate. J. Agric. Food Chem..

[37] Savoi S., Herrera J.C., Carlin S., Lotti C., Bucchetti B., Peterlunger E., Castellarin S.D., Mattivi F. (2020). From grape berries to wines: Drought impacts on key secondary metabolites. OENO One.

[38] Spayd S.E., Tarara J.M., Mee D.L., Ferguson J.C. (2002). Separation of sunlight and temperature effects on the composition of *Vitis vinifera* cv. Merlot berries. Am. J. Enol. Vitic..

[39] Tarara J.M., Lee J., Spayd S.E., Scagel C.F. (2008). Berry temperature and solar radiation alter acylation, proportion, and con-centration of anthocyanin in *Merlot grapes*. Am. J. Enol. Vitic..

[40] Grimplet J., Deluc L.G., Tillett R.L., Wheatley M.D., Schlauch K.A., Cramer G.R., Cushman J.C. (2007). Tissue-specific mRNA expression profiling in grape berry tissues. BMC Genom..

[41] Kennedy J.A., Matthews M.A., Waterhouse A.L. (2002). Effect of maturity and vine water status on grape skin and wine fla-vonoids. Am. J. Enol. Vitic..

[42] Castellarin S.D., Matthews M.A., di Gaspero G., Gambetta G.A. (2007). Water deficits accelerate ripening and induce changes in gene expression regulating flavonoid biosynthesis in grape berries. Planta.

[43] Intrigliolo D.S., Castel J.R. (2009). Response of grapevine cv. ‘Tempranillo’ to timing and amount of irrigation: Water relations, vine growth, yield and berry and wine composition. Irrig. Sci..

[44] Roby G., Harbertson J.F., Adams D.A., Matthews M.A. (2004). Berry size and vine water deficits as factors in winegrape composition: Anthocyanins and tannins. Aust. J. Grape Wine Res..

[45] Martínez-Lüscher J., Brillante L., Kurtural S.K. (2019). Flavonol Profile Is a Reliable Indicator to Assess Canopy Architecture and the Exposure of Red Wine Grapes to Solar Radiation. Front. Plant Sci..

[46] Luzio A., Bernardo S., Correia C., Moutinho-Pereira J., Dinis L.-T. (2021). Phytochemical screening and antioxidant activity on berry, skin, pulp and seed from seven red Mediterranean grapevine varieties (*Vitis vinifera* L.) treated with kaolin foliar sunscreen. Sci. Hortic..

[47] Cerda-Carrasco A., López-Solís R., Nuñez-Kalasic H., Peña-Neira Á., Obreque-Slier E. (2014). Phenolic composition and antioxidant capacity of pomaces from four grape varieties (*Vitis vinifera* L.). J. Sci. Food Agric..

[48] Gironés-Vilaplana A., Moreno D.A., Garcia-Viguera C. (2014). Phytochemistry and biological activity of Spanish Citrus fruits. Food Funct..

[49] Zacarías-García J., Rey F., Gil J.-V., Rodrigo M.J., Zacarías L. (2020). Antioxidant capacity in fruit of Citrus cultivars with marked differences in pulp coloration: Contribution of carotenoids and vitamin C. Food Sci. Technol. Int..

[50] Zarrouk O., Francisco R., Pintó-Marijuan M., Brossa R., Santos R.R., Pinheiro C., Costa J., Lopes C., Chaves M.M. (2012). Impact of irrigation regime on berry development and flavonoids composition in Aragonez (Syn. Tempranillo) grapevine. Agric. Water Manag..

[51] Genebra T., Santos R.R., Francisco R., Pinto-Marijuan M., Brossa R., Serra A.T., Duarte C.M.M., Chaves M.M., Zarrouk O. (2014). Proanthocyanidin Accumulation and Biosynthesis Are Modulated by the Irrigation Regime in Tempranillo Seeds. Int. J. Mol. Sci..

[52] Kennedy J.A., Troup G.J., Pilbrow J.R., Hutton D.R., Hewitt D., Hunter C.R., Ristic R., Iland P.G., Jones G.P. (2000). Development of seed polyphenols in berries from *Vitis vinifera* L. cv. Shiraz. Aust. J. Grape Wine Res..

[53] Castellarin S.D., Pfeiffer A., Sivilotti P., Degan M., Peterlunger E., Di Gaspero G. (2007). Transcriptional regulation of anthocyanin biosynthesis in ripening fruits of grapevine under seasonal water deficit. Plant Cell Environ..

[54] Tkacz K., Wojdyło A., Turkiewicz I.P., Nowicka P. (2020). Anti-diabetic, anti-cholinesterase, and antioxidant potential, chemical composition and sensory evaluation of novel sea buckthorn-based smoothies. Food Chem..

[55] González-Tejedor G.A., Martínez-Hernández G.B., Garre A., Egea J.A., Fernandez P.S., Artés-Hernández F. (2017). Quality Changes and Shelf-Life Prediction of a Fresh Fruit and Vegetable Purple Smoothie. Food Bioprocess Technol..

[56] Jara-Palacios M.J., Gonçalves S., Heredia F.J., Hernanz D., Romano A. (2020). Extraction of Antioxidants from Winemaking Byproducts: Effect of the Solvent on Phenolic Composition, Antioxidant and Anti-Cholinesterase Activities, and Electrochemical Behaviour. Antioxidants.

[57] Teleszko M., Wojdyło A. (2013). Bioactive compounds vs. organoleptic assessment of ‘smoothies’-type products prepared from selected fruit species. Int. J. Food Sci. Technol..

[58] Wojdyło A., Samoticha J., Chmielewska J. (2020). Effect of different pre-treatment maceration techniques on the content of phenolic compounds and color of Dornfelder wines elaborated in cold climate. Food Chem..

[59] Karaman H.T., Küskü D.Y., Söylemezoğlu G. (2021). Phenolic compounds and antioxidant capacities in grape berry skin, seed and stems of six winegrape varieties grown in Turkey. Acta Sci. Pol. Hortorum Cultus.

[60] Xu C., Zhang Y., Cao L., Lu J. (2010). Phenolic compounds and antioxidant properties of different grape cultivars grown in China. Food Chem..

[61] Nowicka P., Wojdyło A., Samoticha J. (2016). Evaluation of phytochemicals, antioxidant capacity, and antidiabetic activity of novel smoothies from selected Prunus fruits. J. Funct. Foods.

[62] Matos M.S., Romero-Díez R., Álvarez A., Bronze M.R., Rodríguez-Rojo S., Mato R.B., Cocero M.J., Matias A.A. (2019). Polyphenol-Rich extracts obtained from winemaking waste streams as natural ingredients with cosmeceutical potential. Antioxidants.

[63] Faria A., Calhau C., Freitas V., Mateus N. (2006). Procyanidins as Antioxidants and Tumor Cell Growth Modulators. J. Agric. Food Chem..

[64] Hidalgo M., Sanchez-Moreno C., de Pascual-Teresa S. (2010). Flavonoid–flavonoid interaction and its effect on their antioxidant activity. Food Chem..

